# A Transdiagnostic Comparison of Emotional Regulation, Executive Functions, and Empathy in Three Groups of Female Adolescents: With Anorexia Nervosa, Attention Deficit Hyperactivity Disorder, and Comorbid Attention Deficit Hyperactivity Disorder and Autism Spectrum Disorder

**DOI:** 10.3390/brainsci16070676

**Published:** 2026-06-27

**Authors:** Francesca Olzi, Daniela Raucci, Antonio Narzisi, Elena Valente, Francesca Ditaranto, Vittorio Belmonti, Raffaella Tancredi, Chiara Pfanner, Emanuela Inguaggiato, Arianna Villafranca, Francesca Lenzi, Stefano Berloffa, Greta Tolomei, Valentina Viglione, Gabriele Masi, Annarita Milone, Pamela Fantozzi

**Affiliations:** 1 IRCCS Fondazione Stella Maris, Viale del Tirreno 331, 56128 Pisa, Italy; francesca.olzi@fsm.unipi.it (F.O.); daniela.raucci@fsm.unipi.it (D.R.); antonio.narzisi@fsm.unipi.it (A.N.); elena.valente@fsm.unipi.it (E.V.); francesca.ditaranto@fsm.unipi.it (F.D.); vittorio.belmonti@fsm.unipi.it (V.B.); raffaella.tancredi@fsm.unipi.it (R.T.); chiara.pfanner@fsm.unipi.it (C.P.); emanuela.inguaggiato@fsm.unipi.it (E.I.); arianna.villafranca@fsm.unipi.it (A.V.); francesca.lenzi@fsm.unipi.it (F.L.); stefano.berloffa@fsm.unipi.it (S.B.); greta.tolomei@fsm.unipi.it (G.T.); valentina.viglione@fsm.unipi.it (V.V.); gabriele.masi@fsm.unipi.it (G.M.); pamela.fantozzi@fsm.unipi.it (P.F.); 2 Department of Clinical and Experimental Medicine, University of Pisa, 56126 Pisa, Italy

**Keywords:** anorexia nervosa, attention deficit hyperactivity disorder, autism spectrum disorder, comorbidity, neurodevelopmental disorders, emotional dysregulation, executive functioning, empathy, female adolescents

## Abstract

**Background**: Anorexia Nervosa (AN) is a severe eating disorder. Attention Deficit Hyperactivity Disorder (ADHD) and Autism Spectrum Disorder (ASD) are two Neurodevelopmental Disorders (NDDs), frequently co-occurring with each other (ADHD+ASD). The present study aimed to clarify cognitive and behavioral profiles, with a specific focus on emotional regulation, executive functions and empathy, in three groups of female adolescents. **Methods**: A total of 102 female adolescents aged 12–18 years were recruited. Participants were divided into three groups (AN: *n* = 30, ADHD: *n* = 47, ADHD+ASD: *n* = 25). All participants underwent a psychometric and a multidimensional clinical assessment. Group differences were analyzed through ANOVA with Bonferroni corrections. **Results**: Adolescents with ADHD+ASD scored significantly higher than the ADHD group in verbal comprehension. The AN group performed significantly better than both the ADHD and ADHD+ASD groups in working memory, and significantly better than the ADHD+ASD group in processing speed. Both the AN and ADHD+ASD groups were characterized by significantly greater impairment in global functioning than the ADHD group. No significant differences were found among the three groups on the Attention Switching, Attention to Detail, and Imagination subscales of the Autism Spectrum Quotient. Behaviorally, AN participants exhibited higher internalizing symptoms (anxiety and depression), the ADHD group presented more prominent externalizing behaviors (aggressive, rule-breaking, and attention problems), and the comorbid ADHD+ASD group demonstrated significantly more pronounced social problems. Most measures used to assess emotional dysregulation did not reveal significant differences among the three groups. Both the ADHD and ADHD+ASD groups showed significantly greater impairment in executive functioning than the AN group. Regarding empathic abilities, mixed results emerged. **Conclusions**: Findings suggest the coexistence of condition-specific features and shared vulnerabilities in female adolescents with AN, ADHD, and ADHD+ASD. These data underscore the importance of investigating the female phenotype from a transdiagnostic perspective to facilitate early detection and tailored interventions.

## 1. Introduction

Anorexia Nervosa (AN) and Neurodevelopmental Disorders (NDDs), such as Attention Deficit Hyperactivity Disorder (ADHD) and Autism Spectrum Disorder (ASD), are classified as distinct diagnostic conditions in the DSM-5-TR [1], although they frequently co-occur. AN is a severe and often chronic Eating Disorder (ED), more common in females than males [2], with a peak incidence during adolescence [3,4]. Its core pathological features include persistent restriction of energy intake and/or purging behavior, an intense fear of weight gain, and a disturbance in the self-perception of one’s body shape [1]. ADHD is a common NDD, with onset typically occurring during infancy. It is characterized by symptoms of inattention, hyperactivity, and impulsivity. In addition, individuals with ADHD frequently exhibit externalizing behaviors, including aggression and rule-breaking tendencies [5,6]. ASD is a pervasive and early-onset NDD, characterized by deficits in social communication and interaction together with restrictive and repetitive patterns of behavior and/or interests [1]. Individuals with ASD also commonly present internalizing symptoms, such as withdrawn and depressive traits [7,8]. The male to female ratio has been estimated at approximately 3-4:1 for ADHD [9] and 4:1 for ASD [10]. However, growing evidence suggests that females with NDDs may present with less overt behavioral manifestation and a greater prevalence of internalizing symptoms, alongside compensatory and camouflaging strategies, which may mask underlying difficulties [11,12]. In this context, the female phenotype refers to a descriptive, sex- and gender-related clinical presentation in which core neurodevelopmental symptoms may be expressed in ways that differ from those typically captured by traditional diagnostic prototypes [13,14]. The recent literature further suggests that the ADHD+ASD comorbidity cannot be considered merely the sum of the diagnostic features of the two disorders, but rather a distinct clinical phenotype, involving both the accumulation and amplification of vulnerabilities associated with each condition. Specifically, individuals with co-occurring ADHD and ASD may exhibit more severe cognitive impairments and broader emotional–behavioral dysregulation compared with subjects with either disorder alone [15]. Moreover, the co-occurrence of ADHD and ASD appears to be highly prevalent, particularly in clinical samples, supporting the view that this dual diagnosis reflects a common clinical rather than an exceptional condition [16].

The term emotion regulation refers to any strategy that modulates emotion unfolding and shapes the resulting reactions, both emotionally and behaviorally [17,18,19]. Emotional dysregulation, to date widely recognized as a transdiagnostic dimension, is characterized by mood lability, emotional reactivity and impulsivity, and overt irritability [20]. It is well documented that emotional symptoms are common and persistent and often cause clinically significant impairments in youth and adults with ADHD [21], as well as in individuals with ASD [22]. At the same time, in recent years, a growing body of literature has identified broad emotion regulation problems in individuals with AN [23,24]. Empathy is a complex multidimensional construct including an affective component, referred to as affective empathy, which is the capacity to share emotions and respond to the emotional displays of others, and a cognitive component, namely cognitive empathy, which is the ability to understand the perspective of another person [25,26,27,28,29]. Although the two systems work independently, they interact with each other [30,31]. Clinical evidence suggests that empathy deficits have been implicated in neurodevelopmental conditions in childhood and adolescence, including ADHD [32,33,34,35,36] and ASD [37,38,39]. Even if findings regarding empathy in AN are mixed, Kerr-Gaffney et al. [40] concluded that the reduced cognitive empathy and intact affective empathy profile found in AN was similar to that of other neurodevelopmental conditions, such as ASD. Empathy and executive functions are multidimensional constructs that enable individuals to cope with their environment. Previous studies have demonstrated a positive correlation between executive functions, emotion regulation, and empathy in healthy subjects [41]: inhibitory control, working memory, and cognitive flexibility were more strongly related to cognitive empathy, while inhibitory control was also closely related to the affective component.

Studies on EDs in NDDs and vice versa indicate a frequent association of these disorders [42] and the presence of shared alterations in executive functioning, particularly in cognitive flexibility, planning, inhibition, and behavioral monitoring [43,44]. However, most works have examined these disorders separately: ASD and ADHD [45], ASD and AN [46,47,48], and ADHD and EDs [49]. As a result, the extent of overlap and the possible presence of specific and stable differences in emotional-affective and behavioral profiles between AN and NDDs in female samples remain poorly characterized. Studying females is therefore relevant not only to ensure adequate representation, but also because adolescent girls may present a combination of internalizing symptoms, emotion-regulation difficulties, socio-cognitive vulnerabilities, and restrictive eating behaviors that may obscure or modify the clinical expression of ADHD and ASD [50,51],. In the last decade, the association between AN and ASD has been widely investigated, both regarding the potential mechanisms underlying this association [50] and the neuropsychological and socio-emotional profiles [52,53]. Research has documented an elevated presence of comorbid autistic symptoms among people with AN [46,48], and overlapping features between AN and ASD, such as alterations in the executive functioning profile [54], difficulties with emotion recognition [55], empathic processing, with an imbalance between cognitive and affective components of empathy [56,57], and mental state understanding [52]. In contrast, only a few studies have investigated similarities and differences between AN and ADHD [42].

Based on this background, the aim of the present study was to examine similarities and differences in cognitive, affective and behavioral profiles, with a specific attention on emotional regulation, executive functions and empathy, in three groups of female adolescents: those with AN, ADHD, and ADHD+ASD. The ADHD+ASD group was considered in the present study not as the mere sum of two diagnostic categories, but as a clinically meaningful comorbid phenotype. The aim was therefore to compare AN, ADHD, and ADHD+ASD profiles from a transdiagnostic perspective, rather than to isolate the independent contribution of ASD or ADHD symptoms. This approach may contribute to a better characterization of the female phenotype, by highlighting clinical features that could support its recognition and potentially inform earlier identification and diagnostic assessment.

## 2. Materials and Methods

We conducted an observational, comparative study. 

### 2.1. Participants

We recruited all female patients who were consecutively admitted to our tertiary care research hospital for child and adolescent neuropsychiatry from February 2024 to September 2025. Eligibility criteria were defined as follows: diagnosis of AN, ADHD, or ADHD+ASD according to the Diagnostic and Statistical Manual of Mental Disorders, 5th edition, Text Revision (DSM-5-TR) criteria [1]; female sex; aged between 12 and 18 years; Full-Scale Intelligence Quotient equal to or greater than 85; and absence of pharmacological treatment at recruitment (in case of previous pharmacological treatment, a washout period of at least three months was required). The exclusion criteria were the same as those used in previous works [23,58]: psychotic symptoms; neurological disorders or sensory deficits; current or history of substance abuse; medical conditions not correlated with the eating disorder; significant intrinsic instability requiring constant medical care supervision.

Informed consent was obtained from all subjects involved in the study and their parents or legal guardians. The study was conducted in accordance with the Declaration of Helsinki and approved by the Institutional Review Board of the Meyer Hospital (Florence, Italy; Regional Pediatric Ethics Committee of Tuscany, code Females23, approval date: 13 February 2024).

Patients underwent a full diagnostic assessment including parent- and self-rated clinical questionnaires and the semi-structured interview Kiddie Schedule for Affective Disorders and Schizophrenia-Present and Lifetime Version [59], administered by trained child neuropsychiatrists. Cognitive functioning was assessed using the Wechsler Intelligence Scale for Children-Fourth Edition (WISC-IV) [60,61] for participants between 12 and 16 years and 11 months, and the Wechsler Adult Intelligence Scale-Fourth Edition (WAIS-IV) [62] for participants older than 16 years and 11 months. Both instruments provide a Full Scale Intelligence Quotient (IQ) and four primary indices: Verbal Comprehension, Perceptual Reasoning, Working Memory, and Processing Speed.

### 2.2. Measures

The following clinician-, parent- and patient-rated scales and questionnaires, some of which were previously used in other studies by our group [58,63], were administered:

#### 2.2.1. Clinician-Rated Scales

- The Clinical Global Impression-Severity (CGI-S) [64]: a global scale completed by the clinician that assesses the overall severity of the disorder, regardless of its psychopathological complexity;

- The Children’s Global Assessment Scale (C-GAS) [65]: a clinician-completed scale used to evaluate the patient’s overall level of functional impairment.

#### 2.2.2. Self-Rated Questionnaires and Task Administered to Patients

- The Eating Attitude Test-26 (EAT-26) [66]: a self-administered questionnaire consisting of 26 items, which assess symptoms and characteristics concerns of eating disorders in adolescents and adults. The EAT-26 generally demonstrates high internal consistency, with Cronbach’s alpha coefficients commonly ranging from 0.83 to 0.91 in both clinical and non-clinical populations;

- The Youth Self Report-for ages 11–18 (YSR 11–18) [67]: a widely used, reliable, and validated self-report measure that assesses behavioral problems across three broadband scales (Internalizing, Externalizing, and Total Problems) and several empirically based syndrome subscales (Anxious/Depressed, Withdrawn/Depressed, Somatic Complaints, Social Problems, Thought Problems, Attention Problems, Rule-Breaking Behavior, and Aggressive Behavior). For the purpose of our study, we also calculated the Dysregulation Profile composite index [67,68]. Previous studies have reported Cronbach’s alpha coefficients of 0.70 or higher for most syndrome subscales, and approximately 0.92 for Internalizing and 0.87 for Externalizing Problems, supporting the instrument’s reliability;

- The Behavior Rating Inventory of Executive Function, Second Edition, Self-Report (BRIEF-2 SR) [69]: a 55-item self-report questionnaire assessing executive and regulatory functioning in everyday life from the adolescent’s perspective. It includes seven factors organized into three indices: the Behavioral Regulation Index (Inhibit and Self-Monitor), the Emotional Regulation Index (Shift and Emotional Control), and the Cognitive Regulation Index (Task Monitor, Working Memory, and Plan/Organize). Previous studies have reported robust internal consistency for the BRIEF-2 Self-Report form, with Cronbach’s alpha coefficients ranging from 0.87 to 0.93;

- The Reactivity, Intensity, Polarity, and Stability-Youth version (RIPoSt-Y) [70]: a 31-item questionnaire aimed at assessing the presence of emotional dysregulation across three main dimensions: Affective Instability, Emotional Reactivity, and Interpersonal Sensitivity. It provides corresponding cutoff scores and has demonstrated good validity and reliability, with good-to-excellent internal consistency;

- The Cyclothymic-Hypersensitive Temperament (CHT): a 22-item self-assessment questionnaire employed to identify cyclothymic temperamental traits in young people aged 10 or older. It includes two main factors: Moodiness/Hypersensitiveness and Impulsiveness/Emotional Dysregulation. The scale has demonstrated good internal consistency, with Cronbach’s alpha of approximately 0.82 [71,72];

- The Interpersonal Reactivity Index (IRI) [27]: a 28-item self-report questionnaire providing a multidimensional assessment of empathy through two cognitive subscales (Perspective Taking [PT] and Fantasy [FS]) and two affective subscales (Empathic Concern [EC] and Personal Distress [PD]). Participants respond to each item using a 5-point Likert scale ranging from −2 (‘does not describe me well’) to +2 (‘does describe me well’). We administered the Italian version of the IRI [73], which has demonstrated satisfactory-to-good internal consistency, with Cronbach’s-alpha values for all subscales above 0.63;

- The Reading the Mind in the Eyes Test (RME) [74]: a social cognition task assessing the ability to infer complex mental states from the eye region of faces. The test consists of 28 items, each presenting a photograph of the eye region accompanied by four mental-state descriptors; participants must select the option that best describes the mental state depicted. The RME has shown acceptable internal consistency (Cronbach’s α = 0.605; maximal weighted reliability = 0.719) and good test–retest stability (ICC = 0.833).

#### 2.2.3. Parent-Rated Questionnaires

- The Autism Spectrum Quotient-Adolescent Version (AQ-Adolescent) [75,76]: a 50-item parent-report questionnaire assessing autistic traits in adolescents aged 12 to 18 years. It includes five domains (Social Skills, Communication, Imagination, Attention to Detail, and Attention Switching) and a total score ranging from 0 to 50. The instrument demonstrates good internal consistency (Cronbach’s α = 0.79) and excellent test–retest reliability (r = 0.92), supporting its clinical utility in adolescent populations;

- The Conners’ Parent Rating Scale-Revised, Short Form (CPRS-R:S) [77]: a standardized parent-report measure designed to assess ADHD symptoms and related behavioral difficulties in children and adolescents. It demonstrated good internal consistency and strong psychometric properties. The short form consists of 27 items rated according to the frequency of the behaviors described and provides age- and sex-normed T scores across four main subscales: Oppositional, Cognitive Problems/Inattention, Hyperactivity, and the ADHD Index;

- The Empathy Quotient-Parent Report (EQ) [78]: a 40-item questionnaire (plus 20 filler items) rated on a 4-point Likert scale, designed to assess empathic abilities by evaluating both cognitive and affective components of empathy. Higher scores indicate greater perceived empathy. The EQ has been shown to be a valid and reliable measure, demonstrating good test–retest reliability and strong construct and concurrent validity.

### 2.3. Assessment Procedures

Participants completed self-report measures individually in a structured clinical setting, whereas parents or primary caregivers completed the parent-report forms. Additionally, a clinician administered the Reading the Mind in the Eyes Test following a standardized procedure. 

### 2.4. Statistical Analyses

Statistical analyses were conducted using IBM SPSS Statistics for Windows, Version 28.0 (IBM Corp., Armonk, NY, USA). Descriptive statistics were calculated for all demographic, clinical, cognitive, and psychopathological variables. Continuous variables were reported as mean ± standard deviation (SD).

Preliminary analyses were performed to examine the distributional properties of the variables and to verify the assumptions for parametric testing. Before conducting one-way ANOVAs, the assumptions of normality and homogeneity of variances were examined. Normality was assessed through visual inspection of histograms and Q-Q plots, as well as by using the Shapiro–Wilk test. Homogeneity of variances was examined using Levene’s test. When this assumption was violated, Welch’s ANOVA was considered as a robustness check. Potential outliers were inspected using standardized values and boxplots. Based on these preliminary checks, parametric analyses were deemed appropriate.

For the main comparative analyses, participants were divided into three groups (AN, ADHD, and ADHD+ASD). Group differences were examined using one-way analyses of variance (ANOVA).

When a significant group difference was detected, post hoc pairwise comparisons were performed using the Bonferroni correction to adjust for multiple comparisons.

Effect sizes were calculated using eta squared (*η*^2^) to estimate the magnitude of group differences. Eta-squared values were interpreted according to conventional benchmarks: η^2^ = 0.01 as small, η^2^ = 0.06 as medium, and η^2^ = 0.14 as large effect size.

Analyses were conducted across multiple domains, including cognitive functioning (WISC-IV/WAIS-IV), clinical severity and global functioning (CGI-S and C-GAS), autistic traits (AQ-Adolescent), general psychopathology (YSR 11–18), emotion regulation (RIPoSt and CHT), attentional and executive functioning (CPRS-R and BRIEF-2 Self-Report), and empathy (EQ, IRI, and RME).

Analyses were conducted on available data for each measure. All statistical tests were two-tailed, and the level of statistical significance was set at *p* < 0.05.

## 3. Results

### 3.1. Demographic and Cognitive Profiles 

We recruited 102 female adolescents aged 12 to 18 years (mean age = 15.2 ± 1.7 years). Participants were divided into three groups (AN: *n* = 30, ADHD: *n* = 47, ADHD+ASD: *n* = 25), which did not differ significantly in age (*p* = 0.15).

We detected significant group differences in the Verbal Comprehension Index (*p* = 0.015) with a medium effect size. Post hoc comparisons indicated that the ADHD+ASD group scored significantly higher than the ADHD group. Significant differences were also found in the Working Memory Index (*p* = 0.004) with a large effect size. Post hoc analyses showed that the AN group performed significantly better than both the ADHD and ADHD+ASD groups. Finally, significant group differences were observed in the Processing Speed Index (*p* = 0.011) with a medium effect size. Post hoc comparisons revealed that the AN group scored significantly higher than the ADHD+ASD group. Further details are shown in Table 1.

### 3.2. Associated Psychopathology and Clinical Features

A significant group effect emerged for the CGI-S (*p* < 0.001) with a large effect size. Both the AN and ADHD+ASD groups showed significantly higher scores than the ADHD group *(p* < 0.001). Similarly, a significant group effect also emerged for the C-GAS (*p* < 0.001) with a large effect size. Post hoc comparisons indicated that both the AN and ADHD+ASD groups scored significantly lower than the ADHD group (*p* = 0.008 and *p* < 0.001, respectively).

Concerning some of the subscales of the AQ, we detected significant group differences with a large effect size. Significant differences emerged for the Social Skills subscale (*p* < 0.001), with the ADHD+ASD group reporting significantly higher scores than both the ADHD and AN groups (*p* = 0.002). Significant differences were also found for the Communication subscale (*p* < 0.001), with the ADHD group scoring significantly higher than the AN group (*p* = 0.040), and the ADHD+ASD group scoring significantly higher than both the ADHD and AN groups (*p* = 0.037 and *p* < 0.001, respectively). Finally, a significant group effect was observed for the AQ total score (*p* < 0.001), with the ADHD+ASD group reporting significantly higher scores than both the ADHD and AN groups (*p* = 0.002 and *p* < 0.001, respectively).

As expected, a significant group effect (*p* < 0.001), with a large effect size, emerged for the EAT-26. The AN group exhibited a significantly greater impairment than both the ADHD and ADHD+ASD groups (*p* < 0.001). 

Concerning the YSR 11-18, we detected significant group differences for both the Internalizing and Externalizing Problems scales. Specifically, for the Internalizing Problems scale (*p* = 0.024), the AN group reported significantly higher scores than the ADHD group (*p* = 0.034). For the Externalizing Problems scale (*p* = 0.001), both the ADHD and ADHD+ASD groups showed significantly higher scores than the AN group (*p* = 0.002 and *p* = 0.011, respectively). Regarding the syndrome subscales, for Anxious/Depressed (*p* = 0.040), the AN group scored significantly higher than the ADHD group (*p* = 0.045); for Withdrawn/Depressed (*p* = 0.004), both the AN and ADHD+ASD groups reported significantly higher scores than the ADHD group (*p* = 0.008 and *p* = 0.039, respectively); for Social Problems (*p* = 0.016), the ADHD+ASD group showed significantly higher scores than both the ADHD and AN groups (*p* = 0.027 and *p* = 0.035, respectively); and for Attention Problems (*p* < 0.001), both the ADHD and ADHD+ASD groups scored significantly higher than the AN group (*p* < 0.001 and *p* < 0.001, respectively). Finally, a significant group effect emerged for the Emotional Dysregulation Index (*p* = 0.011), with the ADHD+ASD group reporting significantly higher scores than the AN group (*p* = 0.009). Effect sizes ranged from medium to large (*η*^2^ = 0.063–0.224).

Regarding the CPRS-R:S, significant group differences were found across all four subscales. Specifically, a significant group effect emerged for both the Cognitive Problems/Inattention subscale (*p* < 0.001) and the ADHD Index (*p* < 0.001), with both the ADHD and ADHD+ASD groups scoring significantly higher than the AN group (*p* < 0.001). Additionally, a significant group effect was observed for the Hyperactivity subscale (*p* = 0.020), with the ADHD+ASD group reporting significantly higher scores than the AN group (*p* = 0.018). For the Oppositional subscale (*p* = 0.019), the ADHD+ASD group showed significantly higher scores than the AN group (*p* = 0.015). Effect sizes ranged from medium to large (*η*^2^ = 0.076–0.294). 

Concerning the BRIEF-2, a significant group effect emerged for the Inhibition scale (*p* = 0.009), with the ADHD group scoring significantly higher than the AN group (*p* = 0.007). Significant differences were also found for the Self-Monitoring (*p* = 0.001), Task-Monitoring (*p* < 0.001), Working Memory (*p* < 0.001), and Plan/Organize (*p* < 0.001) scales. For these variables, both the ADHD and ADHD+ASD groups showed significantly higher scores compared to the AN group (*p* = 0.020 for Self-Monitoring; *p* < 0.001 for the remaining three scales). Effect sizes ranged from small to large (*η*^2^ = 0.028–0.252).

No significant differences were found among the groups for the RIPoSt subscales. 

Regarding the Impulsivity/Emotional Dysregulation factor of the CHT, significant group differences were detected (*p* = 0.004), with a medium effect size. Post hoc comparisons indicated that the ADHD group scored significantly higher than the AN group (*p* = 0.003). 

**Figure 1:**
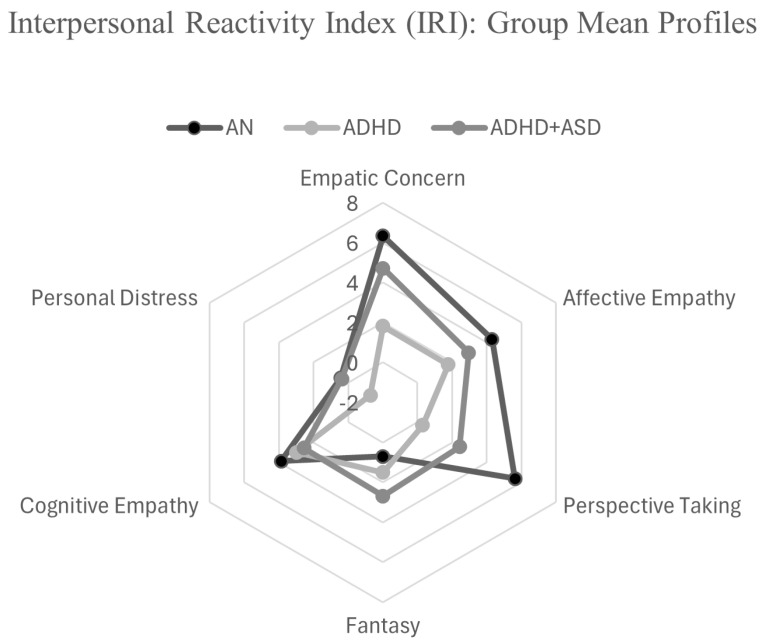
Empathic profiles, as measured by the IRI, among the three groups.

Concerning the EQ, we found significant group differences with a large effect size (*p* < 0.001). Post hoc comparisons revealed that the AN group showed significantly higher mean scores than both the ADHD and ADHD+ASD groups (*p* < 0.001); furthermore, the ADHD group scored significantly higher than the ADHD+ASD group (*p* = 0.005). A significant group effect was also found for two subscales of the IRI: for Perspective Taking (*p* < 0.001), the AN group reported significantly higher scores than the ADHD group (*p* < 0.001); for Personal Distress (*p* = 0.012), the AN group showed significantly higher mean scores than the ADHD+ASD group (*p* = 0.012). Effect sizes ranged from medium to large (*η*^2^ = 0.086–0.149). Figure 1. Finally, no significant differences were observed among the groups for the RME (*p* = 0.328). Further details are shown in Table 2.

## 4. Discussion

This is an exploratory and preliminary study aiming to investigate shared and distinctive characteristics in terms of neuropsychological and psychopathological profiles among three groups of female adolescents: with Anorexia Nervosa (AN), Attention-Deficit/Hyperactivity Disorder (ADHD), and comorbid ADHD and Autism Spectrum Disorder (ADHD+ASD). Patients were consecutively recruited at a tertiary care University hospital.

The ADHD+ASD group scored significantly higher than the ADHD group on the Verbal Comprehension Index (VCI) of the WISC-IV. Our results differ slightly from those of a recent work comparing three groups of children and adolescents with NDDs (ASD, ADHD, and ASD+ADHD) [15], in which no significant differences emerged for the VCI among the groups. Notably, compared to our study, the sample by Narzisi et al. [15] included younger children and only one-sixth of the patients were females. In a recent review examining cognitive abilities in ASD and ADHD, Wilson [79] concluded that the cognitive profile of subjects with autism was characterized by relative strengths in verbal reasoning compared to those with ADHD. Our findings may suggest that individuals with comorbid ADHD and ASD present better verbal abilities than those with ADHD alone. The AN group showed significantly higher scores on the Working Memory Index (WMI) than both the ADHD and ADHD+ASD groups, and on the Processing Speed Index (PSI) than the ADHD+ASD group. These results are in line with previous studies indicating that subjects with comorbid ADHD+ASD present compounded cognitive impairments [15,80]. The WMI assesses the ability to hold and manipulate in mind both verbal and visual information. Brooks et al. [81] hypothesized that higher WM capacity may support the neural processes underlying excessive epistemic foraging—the cognitive sampling of the environment to test predictions about the world—which may reduce distraction from salient stimuli. Conversely, lower WM capacity may be associated with higher impulsivity and reduced epistemic foraging. Based on this model, our findings may suggest that both the ADHD and ADHD+ASD groups experience greater difficulties with short-term memory functioning and increased susceptibility to distraction by salient stimuli compared to the AN group. The PSI evaluates the ability to quickly and accurately scan, discriminate, and order simple visual information. In line with our findings, extant literature indicates that the cognitive profiles of both individuals with ADHD [82] and ASD [79] are characterized by weakness in processing speed.

Both the AN and ADHD+ASD groups showed significantly greater impairment in global functioning compared to the ADHD group, as measured by the clinician-completed CGI-S and C-GAS scales. Clinical severity assessment based on the CGI-S scale revealed marked symptoms severity with high interference in daily life. Similarly, lower mean scores on the C-GAS identified the presence of meaningful deficits in global functioning across almost all areas of daily life, such as social functioning.

Concerning autistic traits, as assessed by the AQ, the ADHD+ASD group exhibited significantly higher scores on the Social Skills subscale and the total score compared to both the ADHD and AN groups. These findings may highlight the greater socio-communicative impairment in the ADHD+ASD group relative to the other two groups, in line with evidence of increased clinical complexity in co-occurring ASD and ADHD [16]. Regarding the Communication subscale, both the ADHD and ADHD+ASD groups scored significantly higher than the AN group; furthermore, the combined ADHD+ASD group also scored higher than the ADHD group. Our results may indicate a gradient of impairment across neurodevelopmental conditions [45,83]. No significant differences were detected among the three groups for the Attention Switching and Attention to Detail subscales, which evaluate the ability to adapt to changes and the tendency to focus strongly on details, respectively. These data may be consistent with evidence of cognitive rigidity in restrictive eating disorders, particularly in acute phases or more severe cases [84,85], supporting the notion of a partial overlap with neurodevelopmental profiles [46,47]. We also found no significant differences in the capacity for imaginative thinking, as measured by the Imagination subscale of the AQ, among the three groups.

As expected, the AN group showed more severe food-related symptoms than the other two groups. Regarding psychiatric comorbidities, as measured by the YSR 11–18, the AN group showed significantly higher levels of internalizing symptoms, such as anxiety and depression, compared to the ADHD group, in line with the most frequent comorbidities reported in individuals with AN [86,87,88]. The ADHD group presented significantly more prominent externalizing behaviors, such as aggressive, rule-breaking, as well as attention problems, than the AN group, in accordance with the typical characteristics of impulsivity and inattention observed in individuals with ADHD [6,89]. The comorbid ADHD+ASD group demonstrated significantly more pronounced social problems than both the AN and ADHD groups, in line with the socio-relational impairment typical of individuals with autism. The combined phenotype also showed significantly higher levels of emotional dysregulation, as evaluated through the composite index of the YSR 11–18, compared to the AN group, in accordance with previous studies documenting greater dysregulation in individuals with combined ASD and ADHD [15,90].

As expected, the two NDD groups scored significantly higher than the AN group on the CPRS-R:S. However, while the combined ADHD+ASD group showed significantly higher scores on all four subscales compared to the AN group, the ADHD group exhibited significantly higher scores only on the Cognitive Problems/Inattention subscale and the ADHD Index. Once again, the comorbid group demonstrated greater impairment in both attention and behavior than the AN group. Concerning the absence of significant differences between the ADHD and AN groups on the Oppositional and Hyperactivity subscales, one possible explanation is that the ADHD group consisted mainly of patients with the inattentive subtype. No significant differences were detected between the two NDD groups on any of the subscales.

Regarding the BRIEF-2, both NDD groups showed greater impairments in executive functioning than the AN group. Previous studies conducted on children and adolescents with NDDs documented significantly impaired executive functions [91,92]. Specifically, the ADHD and ADHD+ASD groups showed significantly higher scores on the Self-Monitor, Task-Monitoring, Working Memory, and Plan/Organize scales compared to the AN group. The ADHD group also showed significantly higher scores on the Inhibit scale than the AN group. One hypothesis is that female patients with ADHD and comorbid ADHD+ASD may exhibit a comparable level of impairment in executive functions. No significant differences were found between groups on the Shift and Emotional Control scales, which comprise the Emotional Regulation Index. The lack of differences on the Shift scale of the BRIEF-2, which measures the ability to transition, solve problems flexibly, and shift focus, between subjects with NDDs and those with AN, appears in line with findings documenting an impairment of some executive functions in AN, particularly cognitive flexibility; this suggests a potential overlap across clinical groups in our samples [84,85]. Similarly, in a recent study of children and adolescents with AN or ASD [54], reported greater impairments in executive functioning in subjects with ASD, as assessed using the parent-report version of the BRIEF. Unlike our study, their sample included both sexes, and the majority of participants with ASD were male. Contrary to expectation, we did not find any significant differences among the three groups on the Emotional Control scale of the BRIEF-2, which evaluates emotional lability, mood change and outburst, or on the RIPoSt-Y. Similarly, no significant differences were detected on the Moodiness/Hypersensitiveness factor or on the total score. We only found a significant difference on the Impulsivity/Emotional Dysregulation factor, which has been shown to be associated with externalizing symptoms [71,72]: the ADHD group showed higher scores than the AN group. The observed pattern may reflect a combination of disorder-specific features within an overall shared profile. In line with recent studies on female adolescents with AN documenting a greater prevalence of emotional dysregulation and cyclothymic traits [23,63], a potential account for these findings is that patients with AN and NDDs may perceive similar levels of impairment in emotion regulation. These data are consistent with the characterization of emotional dysregulation as a transdiagnostic vulnerability factor across neurodevelopmental and psychopathological profiles [71,93].

Concerning the EQ, the AN group showed higher scores compared to both the ADHD and ADHD+ASD groups. Studies using the EQ in patients with AN reported mixed findings [40]; our results may reflect a pattern of increasing impairment in empathic functioning across these neurodevelopmental conditions, as evaluated by parents, in line with previous literature [33,94]. The ADHD group scored higher than the ADHD+ASD group. Our results differed slightly from another recent work by Aiello et al. [95], in which the authors did not find any significant differences in the empathic profiles of two groups of children (ADHD vs. comorbid ADHD+ASD), as measured by the EQ. One possible interpretation is that the differences we observed were partly due to the characteristics of patients recruited: the sample by Aiello et al. [95] was composed entirely of male children aged 6 to 14 years. To date, research evaluating empathic abilities through the IRI has primarily focused on comparing a single disorder to neurotypical populations, while direct transdiagnostic comparisons between distinct clinical groups remain scarce. In the present study, significant group effects emerged for the Perspective Taking and Personal Distress subscales. Specifically, the ADHD group exhibited lower scores on the Perspective Taking subscale compared to the AN group. Previous studies on empathy in ADHD have predominantly focused on male children, documenting low levels of social perspective taking and affective empathy [95,96,97,98]. However, to our knowledge, there are no studies focusing specifically on female adolescents. The AN group showed significantly higher scores on the Personal Distress subscale compared to the ADHD+ASD group. Our results are in line with those by Beadle and Paradiso [99], who compared women with AN during both starvation and weight restoration, finding greater levels of personal distress in the former group. The authors speculated that greater personal distress may be a persistent feature of AN, functioning as a consequence of poor emotional awareness and regulation. At the same time, given that the Personal Distress subscale assesses the tendency to experience discomfort when confronted with others in need [27], another possible explanation is that individuals with AN display a more self-oriented and less regulated form of affective empathy than individuals with ADHD+ASD. No significant differences were found among the groups for the other IRI subscales. Data from literature indicated that individuals with AN often present significantly lower cognitive scores on the IRI than healthy controls, although results remain mixed [40]. In our study, however, the comparison group included adolescents with NDDs rather than healthy subjects. No significant group differences were observed on the RME, suggesting comparable performance in the ability to recognize others’ emotional states across these three groups. In a recent review and meta-analysis evaluating the performance of patients with eating disorders on the RME, Preti et al. [100] found that only patients with active AN showed worse performance than controls, and these data were more likely to be found in earlier studies. In a previous work evaluating empathic abilities via the RME in two groups of children (ASD vs. comorbid ADHD+ASD), Colombi and Ghaziuddin [90] found that the latter group presented the poorest performance.

Several limitations should be considered. Firstly, the relatively small number of patients per group, combined with the large number of statistical tests, may have reduced the statistical power and increased the risk of both type I and type II errors. Therefore, the findings and their clinical implications should be interpreted with caution. Future studies with larger cohorts are needed to replicate these findings and allow for more robust conclusions. Additionally, longitudinal studies are required to assess the evolution of these profiles. Secondly, although the three groups did not significantly differ in age, the relatively broad developmental range of 12–18 years may have influenced executive functioning, emotional regulation, social cognition, and empathy. Future studies with larger samples should control for age more directly, for example by including age as a covariate or by conducting age-stratified analyses. Thirdly, the absence of correlation analyses limited a more nuanced interpretation of the findings. Fourthly, participants were consecutively recruited from a single tertiary care center and were homogeneous in terms of sex. While these aspects represent a notable strength of the study, they also limit the generalizability of the findings to broader clinical settings, such as outpatient populations and male patients. Furthermore, our study did not include a “pure” ASD group or a healthy control group; their inclusion would have allowed for a more detailed comparison across different neurodevelopmental phenotypes and individuals with neurotypical development. The absence of an ASD-only group prevents us from disentangling the specific contribution of autistic traits from the broader ADHD+ASD comorbid phenotype. At the same time, findings related to autistic traits should be interpreted as referring to the ADHD+ASD comorbid phenotype rather than to ASD in isolation. Fifthly, the use of self-report questionnaires to assess eating disorder behaviors and associated psychopathology could result in reporting bias, particularly in a population characterized by limited insight and emotional awareness [101]. Nonetheless, the combination of self-report and parent-report measures, integrated with clinical assessments including semi-structured interviews, enabled a multi-informant and multidimensional investigation of the participants. Therefore, future studies incorporating objective or observational measures are needed to validate the current data.

## 5. Conclusions

Findings from this exploratory study suggest both shared and disorder-specific features across AN, ADHD, and combined ADHD+ASD in female adolescents. Some autistic traits were observed across all three groups. While subjects with NDDs appeared to show greater difficulties in socio-communicative and executive domains, measures of emotion regulation and empathy revealed some overlap across the three groups. Notably, the inclusion of the ADHD+ASD group may have contributed to a more refined characterization of this comorbid phenotype, offering preliminary insights into its clinical and neuropsychological profile. Furthermore, the exclusive focus on female adolescents may represent a further strength, considering that neurodevelopmental conditions can present with distinct and less typical phenotypic features in young women. These findings may indicate that a dimensional and transdiagnostic perspective could represent a useful framework for understanding female population with AN and NDDs, potentially informing assessment strategies and future evidence-based intervention protocols. 

## Figures and Tables

**Table 1:** Age and cognitive profiles of the three groups.

	AN (*n* = 30)	ADHD (*n* = 47)	ADHD+ASD (*n* = 25)	F	*p*	ƞ^2^	Group Significance
**Age**	15.6 ± 1.51	15.1 ± 1.85	14.7 ± 1.68		0.15	-	ns
**VCI**	107.37 ± 13.3	101.22 ± 12.4	111.75 ± 18.7	4.412	0.015	0.087	ADHD+ASD > ADHD
**PRI**	113.22 ± 15.3	107.20 ± 14.2	111.92 ± 19.4	1.394	0.253	0.029	ns
**WMI**	101.27 ± 13.9	88.93 ± 15.8	88.50 ± 17.7	5.833	0.004	0.116	AN > ADHD; AN > ADHD+ASD
**PSI**	100.41 ± 11.6	91.27 ± 19.1	85.54 ± 19.6	4.756	0.011	0.094	AN > ADHD+ASD
**FSIQ**	108.19 ± 13.9	99.97 ± 13.2	104.10 ± 24.7	1.732	0.184	0.043	ns

FSIQ = Full Scale Intelligence Quotient; PRI = Perceptual Reasoning Index; PSI = Processing Speed Index; VCI = Verbal Comprehension Index; WMI = Working Memory Index.

**Table 2:** Clinical features of the three groups.

	AN (*n* = 30)	ADHD (*n* = 47)	ADHD+ASD (*n* = 25)	F	*p*	η^2^	Group Significance
**CGI-S**	4.43 ± 0.7	3.55 ± 0.5	4.60 ± 0.7	27.104	<0.001	0.354	AN > ADHD; ADHD+ASD > ADHD
**C-GAS**	55.77 ± 9.4	61.49 ± 6.8	50.80 ± 7.9	15.292	<0.001	0.204	ADHD > AN; ADHD > ADHD+ASD
**AQ-Adolescent**							
Social Skills	3.63 ± 2.2	3.85 ± 2.5	6.04 ± 2.8	7.498	<0.001	0.132	ADHD+ASD > ADHD; ADHD+ASD > AN
Attention Switching	5.47 ± 1.7	5.68 ± 1.8	6.52 ± 1.6	2.632	0.077	0.050	ns
Attention to Detail	3.93 ± 1.1	3.36 ± 2.0	4.44 ± 2.1	2.962	0.056	0.056	ns
Communication	2.10 ± 1.9	3.40 ± 2.3	4.80 ± 2.2	10.182	<0.001	0.171	ADHD > AN; ADHD+ASD > ADHD; ADHD+ASD > AN
Imagination	2.50 ± 1.7	3.38 ± 2.1	3.40 ± 1.7	2.235	0.112	0.043	ns
**EAT-26**	37.53 ± 22.0	10.13 ± 8.8	9.48 ± 12.5	37.565	<0.001	0.431	AN > ADHD; AN > ADHD+ASD
**YSR-11/18**							
Anxious/Depressed	74.90 ± 11.3	67.38 ± 12.8	72.44 ± 15.0	3.319	0.040	0.063	AN > ADHD
Withdrawn/Depressed	71.27 ± 15.0	62.06 ± 10.9	70.04 ± 12.8	5.890	0.004	0.106	AN > ADHD; ADHD+ASD > ADHD
Somatic Complaints	60.57 ± 8.2	60.40 ± 9.7	62.08 ± 12.9	0.237	0.790	0.005	ns
Social Problems	63.80 ± 10.4	64.19 ± 10.9	71.56 ± 12.3	4.289	0.016	0.080	ADHD+ASD > ADHD; ADHD+ASD > AN
Thought Problems	61.57 ± 13.9	63.30 ± 11.3	68.12 ± 10.5	2.166	0.120	0.042	ns
Attention Problems	60.07 ± 7.5	71.02 ± 10.5	73.28 ± 10.6	15.616	<0.001	0.240	ADHD > AN; ADHD+ASD > AN
Rule-Breaking Problems	53.13 ± 3.8	59.04 ± 7.6	57.24 ± 8.7	6.468	0.002	0.116	ADHD > AN
Aggressive Behavior	54.77 ± 4.4	62.85 ± 9.5	63.48 ± 10.9	9.498	<0.001	0.161	ADHD > AN; ADHD+ASD > AN
Composite Index							
Dysregulation Profile	189.73 ± 16.8	201.26 ± 24.3	209.20 ± 29.0	4.754	0.011	0.088	ADHD+ASD > AN
Internalizing problems	70.17 ± 9.3	63.34 ± 11.9	68.68 ± 12.1	3.869	0.024	0.072	AN > ADHD
Externalizing problems	52.37 ± 5.3	60.45 ± 10.3	60.16 ± 11.9	7.288	0.001	0.128	ADHD > AN; ADHD+ASD > AN
Total Problems	63.03 ± 7.2	64.49 ± 10.2	68.20 ± 11.3	2.019	0.138	0.039	ns
**CPRS-R:S**							
Oppositional	53.27 ± 16.6	60.32 ± 20.4	67.12 ± 13.0	4.152	0.019	0.077	ADHD+ASD > AN
Cognitive Problems/Inattention	51.07 ± 16.0	72.87 ± 24.3	80.88 ± 17.7	16.267	<0.001	0.249	ADHD > AN; ADHD+ASD > AN
Hyperactivity	50.33 ± 18.1	54.79 ± 20.7	64.64 ± 15.7	4.068	0.020	0.076	ADHD+ASD > AN
ADHD Index	52.07 ± 16.0	74.74 ± 20.8	81.21 ± 15.7	20.222	<0.001	0.294	ADHD > AN; ADHD+ASD > AN
**BRIEF-2 SR**							
Inhibition	53.93 ± 8.0	62.96 ± 12.1	60.44 ± 16.5	4.901	0.009	0.090	ADHD > AN
Self-Monitoring	48.50 ± 8.1	58.00 ± 12.0	57.13 ± 13.2	7.011	0.001	0.125	ADHD > AN; ADHD+ASD > AN
Flexibility/Shift	62.40 ± 11.1	65.02 ± 11.0	67.56 ± 12.4	1.393	0.253	0.028	ns
Emotional Regulation	61.23 ± 10.2	65.00 ± 11.2	65.72 ± 11.8	1.420	0.246	0.028	ns
Task-Monitoring	56.53 ± 11.5	67.66 ± 10.6	72.32 ± 13.3	14.026	<0.001	0.221	ADHD > AN; ADHD+ASD > AN
Working Memory	52.93 ± 10.1	65.12 ± 9.4	64.05 ± 9.7	14.979	<0.001	0.252	ADHD > AN; ADHD+ASD > AN
Plan/Organize	55.57 ± 10.5	66.28 ± 12.4	68.16 ± 10.4	10.622	<0.001	0.177	ADHD > AN; ADHD+ASD > AN
**RIPoSt-Y**							
Affective Instability	58.57 ± 17.7	57.64 ± 19.4	58.40 ± 17.1	0.028	0.973	0.001	ns
Emotional Reactivity	23.23 ± 8.3	27.83 ± 8.1	26.52 ± 12.8	2.147	0.122	0.042	ns
Interpersonal Sensitivity	33.67 ± 5.4	29.81 ± 8.0	30.92 ± 8.6	2.400	0.096	0.046	ns
**IRI**							
Empathic Concern	6.33 ± 7.2	1.81 ± 8.4	4.72 ± 8.4	3.045	0.052	0.153	ns
Personal Distress	0.43 ± 4.0	−1.28 ± 5.6	0.36 ± 7.0	4.630	0.012	0.086	AN > ADHD+ASD
Affective Empathy	4.30 ± 4.2	1.77 ± 7.0	2.96 ± 7.7	1.380	0.256	0.020	ns
Perspective Taking	5.63 ± 4.1	0.30 ± 5.9	2.44 ± 5.9	8.663	<0.001	0.149	AN > ADHD
Fantasy	0.70 ± 5.2	1.51 ± 5.0	2.68 ± 4.6	1.053	0.353	0.021	ns
Cognitive Empathy	3.87 ± 2.0	3.00 ± 2.9	2.56 ± 2.8	1.751	0.179	0.034	ns
**RME**	20.17 ± 2.3	18.36 ± 3.1	17.40 ± 4.9	1.128	0.328	0.086	ns

AQ-Adolescent = Autism Spectrum Quotient-Adolescent version; BRIEF-2 SR = Behavior Rating Inventory of Executive Function, Second Edition Self-Report; C-GAS = Children’s Global Assessment Scale; CGI-S = Clinical Global Impression-Severity; CHT = Cyclothymic-Hypersensitive Temperament; CPRS-R:S = Conners’ Parent Rating Scale–Revised: Short Form; EAT-26 = Eating Attitude Test-26; RIPoSt-Y = Reactivity, Intensity, Polarity, and Stability-Youth version; YSR-11/18 = Youth Self-Report for ages 11–18; EQ = Empathy Quotient; IRI = Interpersonal Reactivity Index; RME = Reading the Mind in the Eyes Test.

## Data Availability

The data presented in this study are available on request from the corresponding author.
